# Evaluation of NK Cell Function by Flowcytometric Measurement and Impedance Based Assay Using Real-Time Cell Electronic Sensing System

**DOI:** 10.1155/2013/210726

**Published:** 2013-10-23

**Authors:** Ki-Hyun Park, Hyesun Park, Myungshin Kim, Yonggoo Kim, Kyungja Han, Eun-Jee Oh

**Affiliations:** ^1^Department of Biomedical Science, Graduate School, Catholic University of Korea, 505 Banpo-Dong, Seocho-Ku, Seoul 137-701, Republic of Korea; ^2^Department of Laboratory Medicine, Seoul St. Mary's Hospital, Catholic University of Korea, 505 Banpo-Dong, Seocho-Ku, Seoul 137-701, Republic of Korea

## Abstract

Although real-time cell electronic sensing (RT-CES) system-based natural killer (NK) cytotoxicity has been introduced, it has not been evaluated using human blood samples. In present study, we measured flowcytometry based assay (FCA) and RT-CES based NK cytotoxicity and analyzed degranulation activity (CD107a) and cytokine production. In 98 healthy individuals, FCA with peripheral blood mononuclear cells (PBMCs) at effector to target (E/T) ratio of 32 revealed 46.5 ± 2.6% cytolysis of K562 cells, and 23.5 ± 1.1% of NK cells showed increased degranulation. In RT-CES system, adherent NIH3T3 target cells were resistant to basal killing by PBMC or NK cells. NK cell activation by adding IL-2 demonstrated real-time dynamic killing activity, and lymphokine-activated PBMC (E/T ratio of 32) from 15 individuals showed 59.1 ± 6.2% cytotoxicity results after 4 hours incubation in RT-CES system. However, there was no significant correlation between FCA and RT-CES cytotoxicity. After K562 target cell stimulation, PBMC produced profound proinflammatory and immunoregulatory cytokines/chemokines including IL-2, IL-8, IL-10, MIP-1**α** **β**, IFN-**γ**, and TNF-**α**, and cytokine/chemokine secretion was related to flowcytometry-based NK cytotoxicity. These data suggest that RT-CES and FCA differ in sensitivity, applicability and providing information, and further investigations are necessary in variable clinical conditions.

## 1. Introduction

Natural killer (NK) cells are a major component of innate immunity and are responsible for immune surveillance. They induce direct cytotoxicity or secretion of cytokine/chemokine without recognizing a specific antigen as B and T cells [1]. NK cytotoxicity plays an important role in immune response against infected cells, malignancy, and stressed cells, and involves in pathologic process in various diseases [2–5]. These cytotoxic functions are markedly variable among individuals, and NK function analysis has become a more routine practice in many diseases [6–8].

In clinical laboratory, several methods have been used to define the NK function in different diseases [9–14]. The ^51^chromium release assay has been considered as the gold standard method for measuring NK activity, but it has numerous limitations, such as radioactivity, interlaboratory variability, and low sensitivity to apoptotic cell death [12]. These limitations prevented it from becoming routine test in clinical setting and introduced many alternative methods, including flowcytometry-based NK cytotoxicity assay (FCA). FCA has several advantages such as discrimination of target cells from effector cells and of dead ones from live target cells, enumeration of NK subsets, and possibility of large number of tests. Several studies reported higher sensitivity and reproducibility of FCA versus ^51^chromium release assay [6, 12, 13, 15]. FCA can also measure the specific NK activation markers in addition to the analysis of target cells cytotoxicity. Since strong correlation between CD107a surface expression and NK cytotoxicity was reported [9, 16, 17] and NK function was influenced by cytokine secretion, simultaneous assessment of CD107a and cytokine/chemokine production could be helpful for the complete analysis of NK function. However, FCA results are largely dependent on test variability, including used target or effector cell type and procedures, and limited data is available for healthy individuals [18–20].

The real-time cell electronic sensing (RT-CES) system using xCELLigence (Roche Diagnostics, Penzberg, Germany) has been introduced as an alternative for label-free *in vitro* quantification of NK cell-mediated cytotoxicity [14]. The RT-CES system is microelectronic sensor-based platform integrated into the bottom of microtiter plates, which measure any changes to the cell number, size, morphology, or attachment quality of adherent cells in real time. If target cells are adhered to the culture plate bottom that is coated with the gold microelectrodes, the electrical impedance occurs and is converted to the cell index. In NK function test, when effector cells are added to growing adherent target cells, the cell index decreases and can be changed into the NK cytotoxicity, as it has been used previously for cytotoxic function of NK cell lines on several tumor cell lines [14]. However, to our knowledge, RT-CES based NK cytotoxicity has not been evaluated using human blood samples, and there was no comparative analysis with FCA.

In this study, we tested NK cytotoxicity with peripheral blood mononuclear cells (PBMC) and isolated NK cells from healthy individuals, using flowcytometry and RT-CES system. Also, we analyzed CD107a expression on NK cells and cytokine/chemokine production after K562 target cell stimulation. The purpose of the present study is to evaluate and compare the NK cytotoxicity results from two methods and to investigate different effector/target cells affecting the test results for the routine clinical laboratory setting.

## 2. Materials and Methods

### 2.1. Cell Lines and Cell Culture

K562 is nonadherent and rounded myelogenous leukemia cells line (ATCC, CCL-243), and NIH3T3 is adherent mouse fibroblast cell line (ATCC, CRL-1658). K562 and NIH3T3 cell lines were cultured in DMEM (Welgene, Daegu, Republic of South Korea) supplemented with 10% FBS and 1% penicillin/streptomycin (Gibco, Grand Island, NY) and were used as target cells. NK92 cells (BRC, HC2003) were cultured with *α*MEM media (Gibco, Grand Island, NY) supplemented with 12.5% FBS, 12.5% horse serum, 0.2 mM Myo-inositol (Sigma-Aldrich, St Louis, MO), 0.1 mM 2-mercaptoethanol (Sigma-Aldrich, St Louis, MO), 0.02 mM folic acid (Sigma-Aldrich, St Louis, MO), and 100 U/mL recombinant human IL-2 (Miltenyi-Biotec, Bergisch Gladbach, Germany).

### 2.2. Preparation of Effector Cells

Peripheral blood from healthy donors was collected in heparin tubes and processed within 4 hours. PBMC was isolated by Ficoll-Hypaque density gradient centrifugation. NK cells were separated from PBMC using the human CD56 positive selection kit (human CD56 EasySep positive selection kit, Stemcell Technologies, Vancouver, BC) according to the manufacturer's instructions. PBMC and isolated NK cells were suspended in DMEM media and used in assays without delay. To generate the lymphokine-activated killer (LAK) cells, isolated PBMC and NK cells were stimulated with 6,000 U/mL of human IL-2 (MACS; Miltenyi Biotec, Auburn, CA) for 24 hours. LAK cells were washed and resuspended in DMEM media and used for the NK cytotoxicity test. This study was approved by the Institutional Review Board of Seoul St. Mary's Hospital.

### 2.3. Flowcytometry-Based NK Cytotoxicity Assay

**Figure 1:**
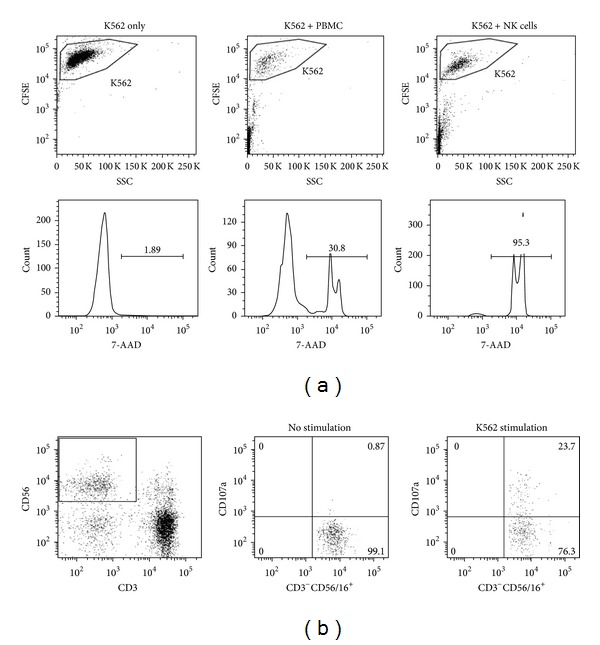
Flowcytometric NK cytotoxicity and CD107a degranulation assay. (a) CFSE labeled K562 cells and effector cells (media only, PBMC, or isolated NK cells) from healthy donors were cultured, and CFSE+7-AAD double labeled K562 cells were analyzed. (b) CD107a expression on NK cells after K562 stimulation was measured to analyze NK cell degranulation.

K562 target cells were labeled with carboxyfluorescein succinimidyl ester (CFSE) (final concentration of 2 *μ*M) to discriminate target cells from effector cells. Then, effector cells (PBMC or NK cells) were incubated with CFSE labeled K562 target cells at different effector-to-target (E : T) ratios from 32 : 1, 16 : 1, and 8 : 1 in 96-well plates. The cells were cultured in 150 *μ*L culture media, and 50,000 target cells were used constantly. The positive control with NK92 cell line was tested with K562 cells at E : T ratio of 32 : 1, and negative control with K562 cells alone was also incubated in each test. After coculture for 4 hours at 37°C, 5% CO_2_, the cell mixture was stained with 5 *μ*L of 7-AAD (Beckman Coulter, Milan, Italy) for 15 min in the dark. Flowcytometry data were analyzed on FACS Fortessa instrument (BD bioscience), using FlowJo version 10.0.6 software (Tree Star, Ashland, OR). NK cytotoxicity (%) was calculated as cells positive for both CFSE and 7-AAD/total CFSE positive cells, after subtracting the spontaneous lysis (%) in negative control (Figure 1(a)).

### 2.4. NK Cytotoxicity Assay by RT-CES System

NIH3T3 cells (10,000 cells in 100 *μ*L of media) were plated to the 16-well E-plate (Roche Applied Science, Indianapolis, IN) and cultured in the DP version of xCELLigence RT-CES system (Roche Diagnostics, Penzberg, Germany) installed in the CO_2_ incubator. After NIH3T3 cells seeding, the increasing impedance was monitored to get the ideal density of target cells and to determine the time to add the effector cells. When cell index reached 1.0 after overnight culture, 50 *μ*L of culture supernatant in the 16-well E-plate was removed, and the effector cells (PBMC, NK or LAK-PBMC) in 50 *μ*L of media were added to the 16-well E-plate. The positive control with NK92 cell line and negative control without the effector cells were tested in each test. The cell mixtures were additionally cultured for 48 hours in the RT-CES. The proliferation or cytotoxicity of NIH3T3 cells was analyzed and plotted using the RTCA software 1.2 (Roche Applied Science, Indianapolis, IN) every 10 minutes in real time. The cytotoxicity of each effector cell was applied to “% of cytolysis = (CI_no effector_ − CI_effector_)/CI_no effector_ × 100”. To evaluate the direct killing activity of the nonstimulated basal effector cells against NIN3T3 cell lines, freshly isolated human PBMC and NK cells were tested. In addition, the cytotoxicity of stimulated effector cells to NIH3T3 cells was investigated by adding IL-2 (500 U/mL) or using prestimulated LAK cells.

### 2.5. CD107a Degranulation Assay

CD107a expression on NK cells was measured to analyze NK cell degranulation as described [16]. 2 × 10^5^ PBMCs were incubated with or without 2 × 10^5^ K562 cells in 200 *μ*L at 37°C, 5% CO_2_. Following a 4-hour culture, cell mixture was stained with monoclonal antibodies against CD45-Pacific blue, CD3-APC-Cy7, CD56/16-PerCP-Cy5, and CD107a-PE (BD Biosciences, San Jose, CA, USA). The lymphocytes were gated as CD45+ cells, and NK cells were further enumerated as CD45+CD3-CD56/16+ cells. To determine the CD107a expression of NK cells, CD107a positive rate of CD3-CD56/16+ NK cells was analyzed (Figure 1(b)).

### 2.6. Assay for Cytokine Production in NK Cytotoxicity

PBMCs from 30 healthy donors were incubated with K562 cells at 32 : 1 ratio in 150 *μ*L culture media for 4 hours at 37°C. Cell mixture was centrifuged (400 ×g for 5 min), and culture supernatant was stored in a −80°C deep freezer until cytokine was tested. The concentrations of chemokines (IL-8, MIP-*α*/*β*, RANTES) and cytokines (IL-2, IL-10, IL-12p70, IL-15, IFN-*γ*, TNF-*α*) in culture supernatant were measured with MILLIPLEX MAP human cytokine/chemokines panel (Millipore, Schwalbach/Ts, Germany) and Luminex 200 instrument (Luminex, Austin, TX, USA). All procedures were performed by manufacturer's instructions.

### 2.7. Statistical Analyses

Statistical analyses were performed using the MedCalc software version 11.5.1.0 (MedCalc, Mariakerke, Belgium). Results are expressed as the mean ± standard error. The between-group differences of cytotoxicity result were compared by Student's *t* tests. The correlation between the results from different methods was analyzed by a Spearman's rank correlation coefficient test. A *P* value of ≤0.05 was considered as statistically significant.

## 3. Results

### 3.1. Flowcytometry-Based NK Cytotoxicity Results

**Figure 2:**
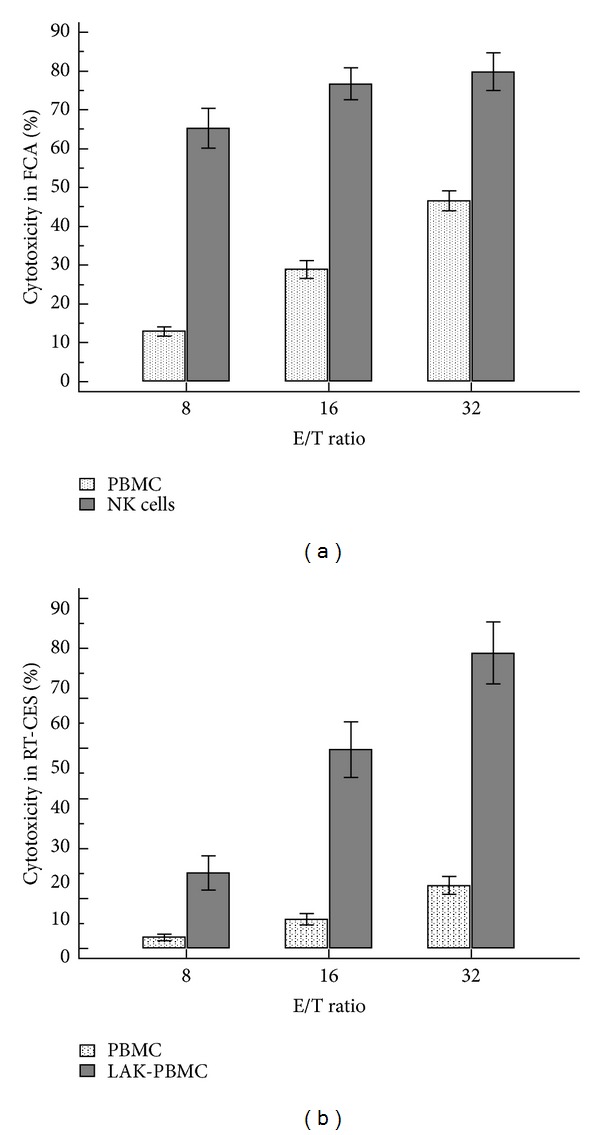
NK cytotoxicity at different E/T ratios. (a) Flowcytometry-based cytolysis of K562 target cells by PBMC (*n* = 98) and isolated NK cells (*n* = 15) from healthy donors. (b) RT-CES system-based cytolysis of NIH3T3 target cells by PBMC (*n* = 46) and lymphokine-activated PBMC (LAK-PBMC) (*n* = 15) from healthy donors.

In the negative control samples, more than 99% of K562 cells were stained with CFSE, and spontaneous lysis was less than 2% of them (Figure 1). NK cell cytotoxicity results with PBMC (*n* = 98) and isolated NK cells (*n* = 15) from healthy donors were shown in Figure 2(a). NK cytotoxicity of PBMC was significantly increased with increasing E : T ratios, and the cytotoxicity levels reached 46.5 ± 2.6% at E/T ratio of 32. The isolated NK cells were much more potent effector cells than PBMC, and all samples showed 65.3 ± 2.6% and 79.4 ± 1.6% cytotoxicity at E/T ratio of 8 and 32, respectively.

### 3.2. Real-Time Profiling of NK Cytotoxicity by RT-CES System

In RT-CES system, the cytotoxicity levels of basal PBMC and NK cells were relatively low, and even different E : T ratios showed little influence on cytotoxicity results. In 46 healthy donors, RT-CES based NIH3T3 cytolysis was 10.3 ± 1.3% after 4 hours stimulation of PBMC at E/T ratio of 32 (Figure 2(b)). 

**Figure 3:**
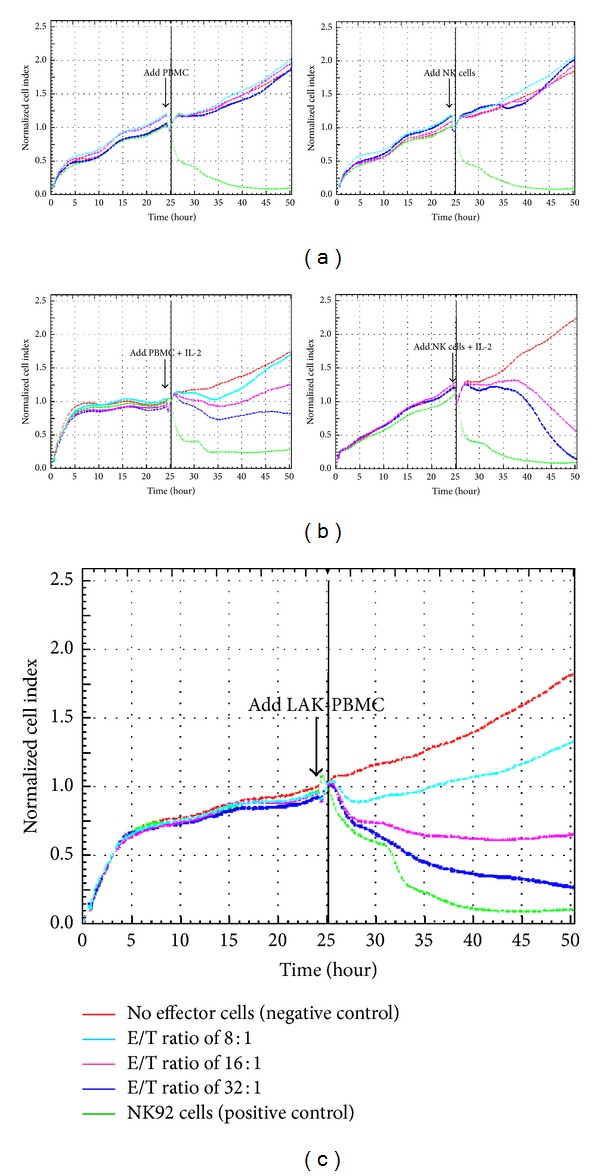
Real-time profiling of NK cytotoxicity by RT-CES system. (a) The NIH3T3 target cells were resistant to basal PBMC and NK cells. (b) Addition of IL-2 induced complete NIH3T3 cytolysis, and complete cytolysis by PBMC and NK cells required more than 72 hours and 24 hours, respectively. (c) LAK-PBMC rapidly killed target cells in 2 hours.

**Figure 4:**
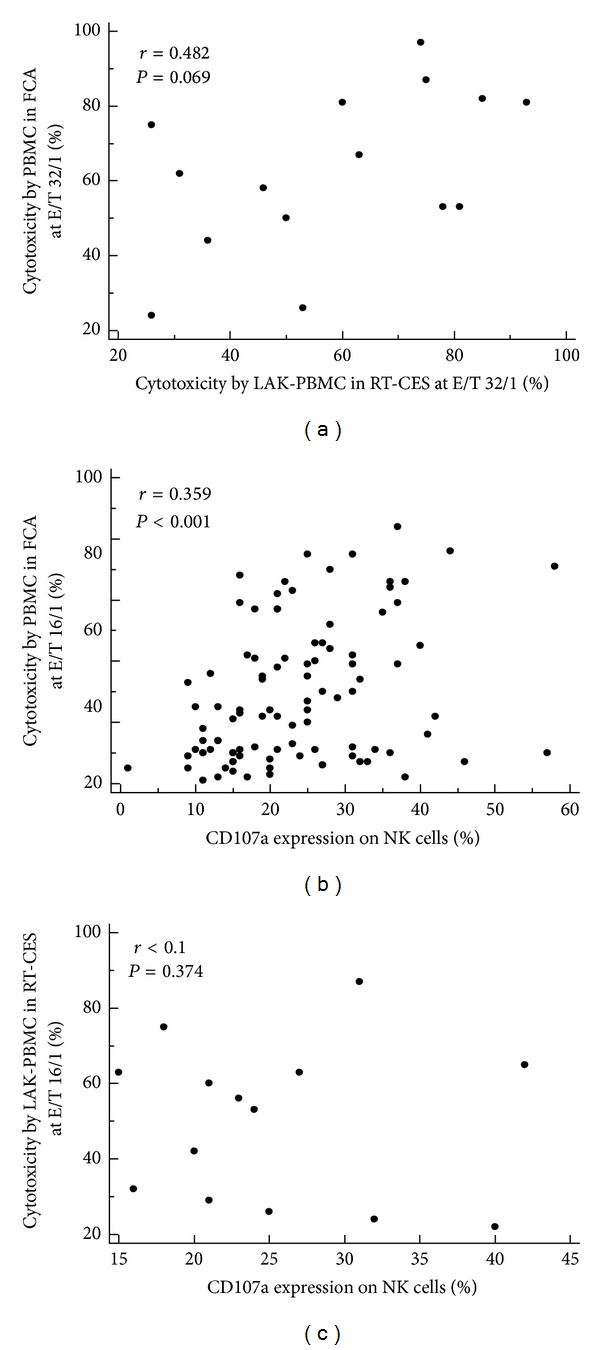
Relation among two NK cytotoxicity tests and CD107a degranulation on NK cells. (a) Correlation between two NK cytotoxicity tests by PBMC in FCA and LAK-PBMC in RT-CES system at E/T ratio of 32. (b) Flowcytometric NK cytotoxicity by PBMC at E/T ratio of 16 versus CD107a expression on NK cells after K562 stimulation. (c) NK cytotoxicity by LAK-PBMC in RT-CES at E/T ratio of 16 versus CD107a expression on NK cells after K562 stimulation.

To reinforce the sensitivity of NIH3T3 cytolysis assay, we applied stimulated effector cells to RT-CES system by adding IL-2 (500 U/mL) or using LAK cells. When NK cells were cocultured with IL-2, complete lysis of NIH3T3 cells by NK cells required about 24 hours, and PBMC required more than 72 hours after adding IL-2 for complete loss of NIH3T3 cells (Figure 3). When we used LAK-PBMC cells, they rapidly killed NIH3T3 target cells in 2 hours after addition of effector cells. In 15 healthy individuals, NK cytotoxicity results by LAK-PBMC (E/T = 32) were 59.1 ± 6.2%. In the comparison between RT-CES using LAK-PBMC and FCA using PBMC, there was no significant correlation between the two methods (*P* > 0.05) (Figure 4(a)).

### 3.3. NK Subsets and CD107a Degranulation on NK Cells

In 98 healthy donors, CD3-CD56/16+ NK cell fraction was 11.1 ± 0.74% of PBMC. The increased NK frequency in PBMC was associated with increased NK cytotoxicity at each E : T ratio (*r* = 0.34–0.36, *P* < 0.001). When the degranulation activity of NK cells was investigated by measurement of CD107a expression, the basal expression of CD107a on NK cells from healthy donors was less than 1%. On stimulation of PBMC by K562 cells for 4 hours, CD107a expression was significantly increased to 23.5 ± 1.1% of CD3-CD56/16+ NK cells (*P* < 0.05). To investigate the relation of CD107a expression with NK cytotoxicity, we analyzed the correlation between the CD107a expression rate of NK cells and FCA results under different E : T ratios. There was a weak but significant correlation between CD107a expression and flowcytometry-based NK cell cytotoxicity using PBMC (*r* = 0.319–0.359, *P* < 0.001) (Figure 4(b)). But no significant correlation was found between CD107a degranulation and RT-CES based NK cytotoxicity using LAK-PBMC (*P* > 0.05) (Figure 4(c)).

### 3.4. Cytokine and Chemokine Production in Culture of PBMC and K562 Target Cells

**Figure 5:**
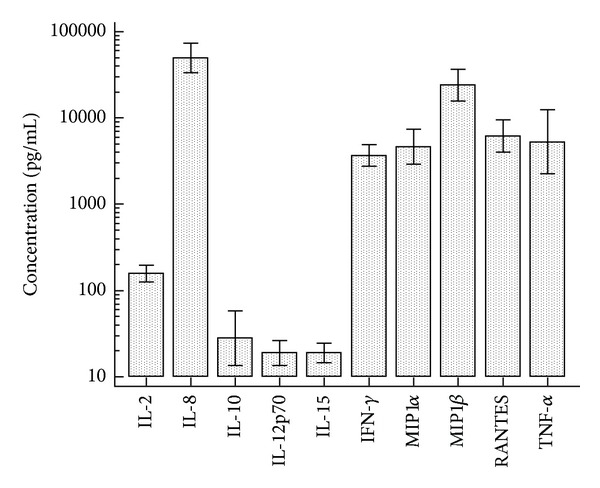
Cytokines/chemokines production after 4 hours incubation of PBMC with K562 cells at E/T ratio of 32. Values represent the mean concentrations from 30 healthy donors.

**Figure 6:**
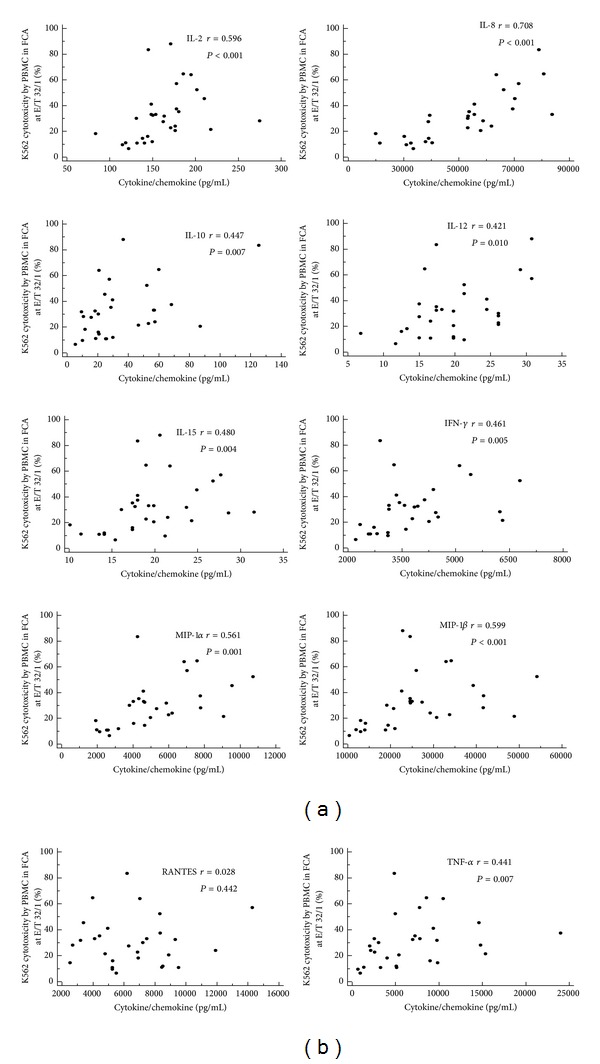
Relation between flowcytometric NK cytotoxicity and cytokine/chemokine secretion after incubation of PBMC with K562 target cells at E/T ratio 32/1.

In 30 healthy donors, cytokine and chemokine productions during incubation of PBMC with K562 were measured. IL-8, MIP-1*β*, RANTES, TN-F*α*, MIP-1*α*, and IFN-*γ* were markedly produced as the concentrations were above 1,000 pg/mL (Figure 5). The association between cytokine/chemokine secretion and NK cytotoxicity results in FCA was analyzed. The concentrations of all studied cytokine/chemokine, except RNATES, showed a significant correlation with NK cytotoxicity activity (*r* = 0.421–0.708, *P* < 0.05) (Figure 6). When we investigate the relationship of cytokine/chemokine production with NK cell number, only IL-15 concentrations were correlated with NK cell numbers.

## 4. Discussion

In clinical laboratory, assessment of NK cell cytotoxicity is increasingly important for the diagnosis and monitoring of the disease progression. In the present study, we investigated both FCA and RT-CES based NK cytotoxicity tests using samples from healthy donors. For FCA, we used K562 cell as the target cell, which lacks the major histocompatibility complex and has been frequently used as target cell for *in vitro* cytotoxicity test in laboratory medicine [21]. With E : T ratio of 32, isolated NK cells from healthy donors showed more than 90% cytotoxicity, and PBMC showed about 40–50% cytotoxicity. Many previous studies reported NK cytotoxicity results using isolated NK cells [16, 22]. However, NK cytotoxicity using PBMC can also provide natural circumstance, like *in vivo* environment as compared to the test using isolated NK cells [21]. When encountering the target cells, the CD56^dim^ cells synchronize secreting the perforin with the granzyme B and then generate the cell cytotoxicity [23]. The activated NK cells can also secrete IFN-*γ* and locally intensify the monocyte and macrophage [24], and the innate and adaptive immune system is reinforced by interaction of the activated NK cells and other immune cells [5]. Thus, in clinical laboratory setting, utilizing PBMC is more convenient and can provide more informative results than utilizing NK cells. We confirmed that NK cells were main IFN-*γ* producing cells in PBMC by the ELISPOT study (data not shown). 

The RT-CES system, using xCELLigence technique, measures electrical impedance from adhesion of living cells and converts it into cell index. It has been used previously for measuring the kinetics of drug in real time, and a few studies for NK cytotoxicity were introduced [25, 26]. RT-CES system measures completely lysed cells, in addition to apoptotic/necrotic dead cells in real time, whereas FCA measures the target cells, which are double-stained with CFSE/7-AAD, and these cells represent only the late apoptotic/necrotic cells with a compromised cell membrane, suggesting that completely lysed necrotic target cells cannot be measured. We investigated the RT-CES system based NK cytotoxicity test, using human PBMC, and demonstrated the effectiveness of it on real time measurement of cytotoxic profiles. When we used basal PBMC from healthy donors as the effector cells without stimulation, cytotoxicity of adherent target cells was little and insensitive to discriminate normal and decreased NK cytotoxicities. It seems that impedance based NK cytotoxicity test using NIH3T3 is less sensitive for human NK effector function analysis.

It is well known that lymphokine-activated NK cells have significantly increased the killing activity compared with the basal NK cells. We confirmed this finding and demonstrated dynamic killing activity of the LAK cells in real-time using the RT-CES system. When we used the effector cells, which were activated by IL-2 before culture, the killing effect on NIH3T3 target cells was immediate, and marked cytotoxicity was demonstrated over the first 2 hours after adding the LAK cells, whereas isolated NK cells with IL-2 showed relatively a slow cytotoxic activity. These findings correspond with previous report in measuring the NK cytotoxicity against the human astrocyte in the RT-CES system [27], in which the authors reported that astrocytes were insensitive to the basal NK cells and were resistant to basal killing by the NK cells. On the contrary, NK cells activated by IL-2 prior to culture rapidly killed astrocytes. In the present study, the cytotoxicity data from LAK-PBMC assay were variable among healthy donors (range 26.0–93.0%), suggesting that the RT-CES system might be used categorizing high or low NK function. However, in comparison of RT-CES with FCA, there was no correlation between the cytotoxicity results from the two methods (*P* > 0.05). It might be due to a difference in the target cells, repertoire of stimulating/activating signals, profile of NK killing, cytokine activation with IL-2, or small sample size. NIH3T3 cells lines are lacking both NK activating receptor ligands and inhibitory ligands, while K562 cells lack inhibitory MHC receptor ligands but express activating receptor ligand, such as MIC and ULPB [28]. Therefore, when using the basal PBMC instead of the LAK cells as effector cells, K562 stimulation in FCA is more effective than the RT-CES system using NIN3T3 cells, whereas if other target cells expressing ligands complementary to stimulating receptors or LAK cell were used, RT-CES system could provide a reliable immune function analysis and further understand the immune mechanism. 

FCA can give information about degranulation and receptor expression. NK cytotoxicity results are dependent on the phenotypic, functional and molecular heterogeneity of NK cells, and immune network. Therefore, simultaneous assessment of NK phenotypes markers, in addition to NK cytotoxicity, will be useful for precise comprehensive interpretation. In the present study, K562 stimulation resulted in a significant increase of CD107a expression on NK cells, and the relationship between CD107a expression and cytotoxicity results was found. These findings support previous data showing that CD107a upregulation correlates with lysis of the target cells [11] and suggest CD107a expression as an additive sensitive marker for NK function determination. However, NK cell cytotoxicity is a stepwise combined process including adhesion, activation, and secretion of lytic granules [29, 30], and CD107a expression may not necessarily correlate to NK cytotoxicity. 

Previous studies demonstrated that NK function was influenced by several cytokines, and specific cytokines/chemokines, were produced in response to NK activation [10, 27, 29–33]. In the present study, K562 target cell stimulation with PBMC produced profound proinflammatory and immunoregulatory cytokines/chemokine, such as IL-2, IL-8, IL-10, MIP-1*αβ*, IFN-*γ*, and TNF-*α*, and significant correlation between cytokine secretion and NK cytolysis was found. These results support a previous report showing that K562 stimulation using human isolated NK cells induced MIP-1*αβ*, IFN-*γ*, and TNF-*α* [10]. However, we used PBMC instead of isolated NK cells for cytokines/chemokine production and could not confirm that which cytokine/chemokine was produced by NK cells. Especially, NK cells have previously been reported that they did not secrete IL-10, and IL-2 upon K562 target cells stimulation [10], and we also confirmed these findings with isolated NK cells (data not shown). Nevertheless, the fact that NK cell cytotoxicity is influenced by cytokines and there are clear synergies between activating receptors and cytokine secretion indicates that the measurement of cytokine production may be useful additive NK function marker and can provide information for the comprehensive analysis of NK function.

## 5. Conclusions

In conclusion, we demonstrated both FCA and RT-CES based NK cytotoxicity results using PBMC from healthy donor. Our study indicates that flowcytometry-based NK cytotoxicity assay using K562 target cells is more sensitive to measures of the cytotoxicity levels than the RT-CES system and can provide associated phenotype expression, while RT-CES system using NIH3T3 adherent cells needs NK cell activation by IL-2 and can be used to demonstrate real-time dynamic killing activity, including early apoptosis and complete lysis. These data suggest that FCA and RT-CES cytotoxicity differ in sensitivity, applicability and providing information. Further studies will be needed to evaluate the sensitivities and usefulness of different NK cytotoxicity tests in variable clinical conditions.

## Abbreviations

NK: Natural killer
RT-CES: Real-time cell electronic sensing
FCA: Flowcytometry-based NK cytotoxicity assay
PBMC: Peripheral mononuclear cell
LAK: Lymphokine-activated killer cell
IL-2: Interleukin 2
MHC: Major histocompatibility complex
MIC: Major histocompatibility complex class I-related chain
ULBP: UL16 binding protein.
